# Liver Segmentation Based on Snakes Model and Improved GrowCut Algorithm in Abdominal CT Image

**DOI:** 10.1155/2013/958398

**Published:** 2013-08-26

**Authors:** Huiyan Jiang, Baochun He, Zhiyuan Ma, Mao Zong, Xiangrong Zhou, Hiroshi Fujita

**Affiliations:** ^1^Software College, Northeastern University, Shenyang 110819, China; ^2^Graduate School of Medicine, Gifu University, Yanagido, Gifu 501-1193, Japan

## Abstract

A novel method based on Snakes Model and GrowCut algorithm is proposed to segment liver region in abdominal CT images. First, according to the traditional GrowCut method, a pretreatment process using K-means algorithm is conducted to reduce the running time. Then, the segmentation result of our improved GrowCut approach is used as an initial contour for the future precise segmentation based on Snakes model. At last, several experiments are carried out to demonstrate the performance of our proposed approach and some comparisons are conducted between the traditional GrowCut algorithm. Experimental results show that the improved approach not only has a better robustness and precision but also is more efficient than the traditional GrowCut method.

## 1. Introduction

With the development of modern computer technology and digital medical equipment, medical image has become an important means for clinical doctors to diagnose diseases. Therefore, clinicians bring forward an upcoming need for computer aided diagnosis (CAD) technique. As an important modern image processing technique liver segmentation becomes an important issue of Liver CAD. The accurate segmentation of diverse tissues in the CT image is not only a necessary premise before extracting features of diseases, but also a basic of the three-dimensional image reconstruction and the medical image visualization.

Image segmentation algorithm can usually be classified into two kinds: fully automated and semiautomated segmentation methods. Fully automated algorithm like the threshold-based method [1, 2], edge-based method [2, 3], clustering method [4, 5], region-based method [6, 7], Markov Random Field (MRF) based method [8–10], Snakes-based method [11], and so forth has been improved constantly. However, no automated algorithm can obtain perfect results for any kind of images. So there are many improved semiautomated methods proposed in the research literatures, such as the interactive image segmentation based on graph cut proposed by Boykov and Jolly [12], the random walker technique proposed by Grady [13], and the intelligent scissor [14] algorithm.

GrowCut [15–20] uses cellular automaton to solve pixel labeling task. It is suitable for image segmentation with any dimensions and fits for both the gray image and the color image. Each automata cell has some labels (in case of binary segmentation—“object,” “background” and “empty”). During the process of automata evolution, some cells capture their neighbors, and then replace their neighbors' labels. The GrowCut algorithm can accurately segment fuzzy regions based on the anatomy knowledge and clinical experience of experts. Besides, the GrowCut algorithm can meet the requirements of real-time processing because of its characteristics of fast speed and simple principle. However, there is still under-segmentation problem for liver segmentation based on this method.

Snakes model [11] is an energy-minimizing spline which is guided by external forces and influenced by image forces that pull it toward features such as lines and edges. It is an important kind of deformable model, which needs a closed curve as a priori knowledge. Although it is immune to noise and pseudoedge, it has a strict demand for an initial contour. If the initial contour is far away from the image edge, it is difficult to obtain a good segmentation result.

In this paper, a novel method for liver segmentation in abdominal CT images is proposed based on Snakes model and GrowCut algorithm. Firstly, a novel energy function for the automata evolution based on the graph theory is proposed. Then, an initial liver region extraction based on the improved GrowCut algorithm is conducted, in which the K-means algorithm is innovatively introduced to accelerate the speed of the improved GrowCut approach. Lastly, the segmentation result of the proposed improved GrowCut algorithm is used as an initial contour for the Snakes model for a precise liver segmentation. In this case, the sensibility problem of the initial contour of Snakes model can be solved efficiently. 

## 2. Image Segmentation Based on Snakes Model and the Improved GrowCut Algorithm

**Figure 1:**
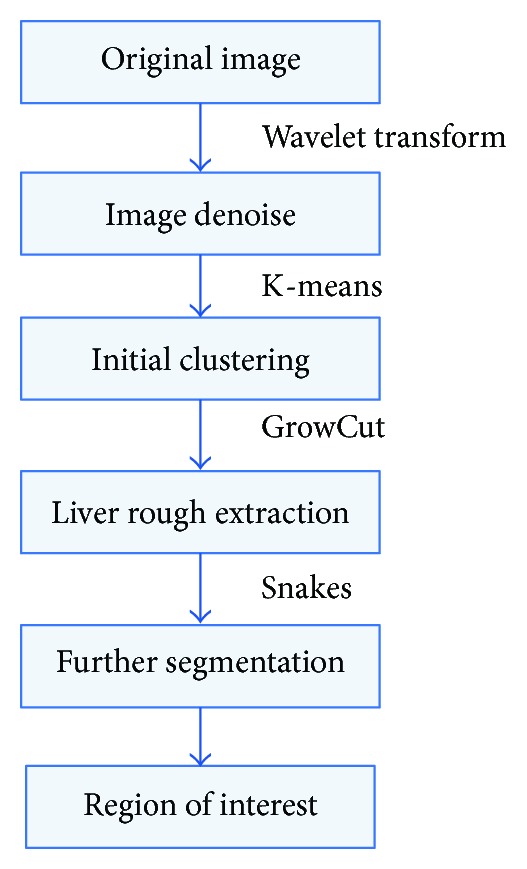
The procedure of the proposed algorithm.

The proposed schema consists of four parts, including image denoise, liver presegmentation, liver region extraction, and liver precise segmentation. Firstly, wavelet decomposition is conducted to denoise an image. Secondly, a liver pre-segmentation based on the K-means algorithm is performed, thirdly, a novel energy function is proposed to improve the GrowCut algorithm and applied in liver region extraction. At the same time, the result of the K-means algorithm is used to accelerate the speed of the improved GrowCut algorithm. Lastly, the segmentation result of the improved GrowCut algorithm is taken as an initial contour for the Snakes model to perform the precise segmentation. The procedure of the proposed algorithm is shown in Figure 1.

### 2.1. Image Denoise Based on Wavelet Transform

Wavelet transform [21] has been widely used in image denoise due to its advantages of time-frequency localization. According to the characteristic of wavelet, the smooth parts in an image are cantered on the low frequency, while the noises and details are distributed in the high frequency. Image denoise methods based on wavelet transform are mainly divided into two kinds: one is directly setting high frequency coefficients as zero and the other is based on a threshold.

After an image is decomposed by wavelet transform, the low frequency component and some high frequency components of an image can be obtained. Compared with the traditional image denoise methods based on a filter, the approach of directly setting high frequency coefficients zero owns a better result and a faster process velocity. However, in the high frequency part of an image, there are not only noises but also some details; such traditional methods usually lead to losing some detail information in an image during the process of wiping out noises in the image.

Let *I* denote an original image, and let *J* denote the decomposition layer; we can get a low frequency component and three high frequency components after three-level wavelet decomposition. So, after decomposing an image, *I* can be described as

(1) $$\begin{array}{c} \left\{I_{2^{3}},I_{2^{j}}^{H},I_{2^{j}}^{V},I_{2^{j}}^{D}\right\}. \end{array}$$

In (1), *I*_2^3^_ denotes the three-level low frequency component; *I*_2^*j*^_^*H*^, *I*_2^*j*^_^*V*^, and *I*_2^*j*^_^*D*^, respectively, denote the *j*-level horizontal, vertical, and diagonal high frequency components. The *j*-level low frequency component of *I* can be characterized as:

(2) $$\begin{array}{c} I_{2^{j}}=\left\{I_{2^{j+1}},I_{2^{j+1}}^{H},I_{2^{j+1}}^{V},I_{2^{j+1}}^{D}\right\}. \end{array}$$

In (2), *I*_2^*j*+1^_ denotes the *j* + 1 level low frequency component, and *I*_2^*j*+1^_^*H*^, *I*_2^*j*+1^_^*V*^, *I*_2^*j*+1^_^*D*^ denote the *j* + 1 level horizontal, vertical and diagonal high frequency components respectively.

Respectively, we use *W*_*y*_*j*__^1^, *W*_*y*_*j*__^2^, and *W*_*y*_*j*__^3^ to indicate the coefficients of three kinds of high frequency in the *j*-level components. The low frequency coefficient of level *j* is signified as *W*_*y*_*j*__^0^. Let *λ* be the threshold. Then the flow of the algorithm is described as follows:

(1) perform the wavelet decomposition for an image, and get the coefficients of each component;
(2) manipulate each high frequency with the following principle:
  (3) $$\begin{array}{c} W_{y_{j}}^{i}=\{\begin{array}{cc} \mathrm{sgn}\left(W_{y_{j}}^{i}\right)*\left[\left|W_{y_{j}}^{i}\right|-\lambda \right], & \left|W_{y_{j}}^{i}\right|\geq \lambda \\ 0 & \left|W_{y_{j}}^{i}\right|<\lambda ; \end{array} \end{array}$$
(3) finally, the denoised image is obtained by taking an inverse wavelet transform.

### 2.2. Image Presegmentation Based on K-Means Algorithm

In fact, there are many tissues and organs distributed in the abdominal CT image and especially the liver attracts the greatest attention. As the gray value of liver differs from skeleton and some other tissues, a clustering operation can be used to wipe out irrelevant tissues and to accelerate the algorithm speed before a segmentation operation. Cluster analysis is a kind of algorithm, which takes the data similarity into account and classifies them. The similarity measurements of medical image are generally gray, distance, and texture. The most common used cluster analysis methods include the K-means [22] and k-Nearest-Neighbours (KNNs) [23] algorithm. From [22, 23], we can know that K-means is very fast and simple, whereas the disadvantage of KNN is its large computation complexity. Considering that we want to use clustering algorithm to improve the efficiency of the whole method, we choose K-means rather than KNN as the classifier. 

K-means algorithm aims to partition *N* observations into number of *K* disjoint subset *S*_*j*_, in which each observation belongs to the cluster with the nearest mean value in order to minimize the sum of squares criterion:

(4) $$\begin{array}{c} J=\sum_{j=1\;}^{K}\sum_{n\in S_{j}}{\left|x_{n}-\mu_{j}\right|}^{2}. \end{array}$$

In (4), *x*_*n*_ is a vector representing the *n*th data point and *u*_*j*_ is the geometric centre of the data points in *S*_*j*_.

**Figure 2:**
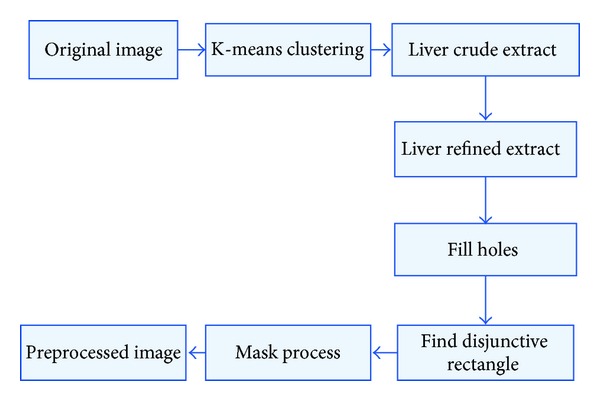
The procedure of image preprocessing by K-means algorithm.

In an abdominal image, there are too many irrelevant regions, which lead to reduction of the processing speed and segmentation precise, so it is necessary to make a cluster analysis before liver extraction using GrowCut algorithm in order to reduce its iteration times. The outline of the process procedure is shown as Figure 2.

**Figure 3:**
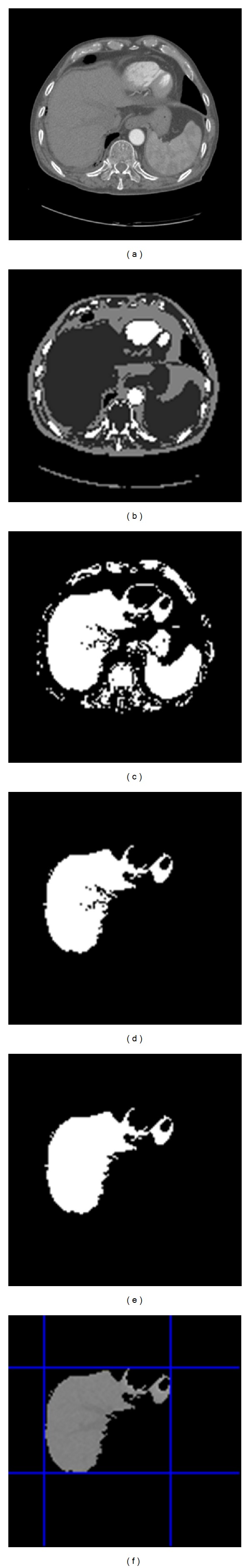
Results of image preprocessing by K-means algorithm. (a) Original image, (b) result image of K-means, (c) liver crude extract image, (d) liver refined extract image, (e) holes filled image and (f) Final preprocessed image.

Firstly, a denoised image is the input of K-means clustering, and then each pixel in the image is labelled. Secondly, the pixels whose labels are the same then regarded as the same region which is extracted finally. Thirdly, the maximum one of all extracted regions is accounted as liver region as it generally takes up the maximum area in an abdominal image; Fourthly, some holes in the candidate liver region are filled and a disjunctive rectangle of the filled candidate liver region is calculated. Lastly, a mask operation is carried out to extract the final result. The result is shown in Figure 3.

In Figure 3, the size of the original image is 128 × 128 and the size of the candidate liver image is 70 × 58. We can efficiently improve the segmentation efficiency and increase the segmentation precision using the K-means preprocessing algorithm.

### 2.3. Liver Region Extraction Based on the Improved GrowCut Approach

Cellular automata (CA) algorithm was introduced by Von Neumann [19], which has been widely used in image denosing and edge detection. A cellular automaton consists of cellular, cellular space, neighbour, and some rules.

The basic cell in CA is called a cellular. A CA is a triplet and is defined as

(5) $$\begin{array}{c} A=\left(S,N,\delta \right). \end{array}$$

Here, *S* is a nonempty state set, *N* is the neighbourhood system and *δ* represents the rule. There are mainly two neighbourhood system models: one is the Von Neumann model and the other is the Moore model. 

The rule is an evolution basis of a dynamic system, which is a dynamic function calculating the state of cellular of the next iteration. 

A digital image is a two-dimensional array with *m* × *n* pixels, and it can be considered as several particular state of CAs, where the cellular space *P* is defined as

(6) $$\begin{array}{c} l_{p}=0,\;\;\theta_{p}=0,\;\;\vec{C_{p}}={\text{RGB}}_{p}. \end{array}$$

Here, RGB_*p*_ is the colour space of pixel *p*, *θ*_*p*_ denotes its intensity, and the final goal is to assign labels to each pixel.

Before the segmentation operation is performed, the user needs to manually input two types of marked points, one-type of points is the foreground (region of interest) points, whose label value *l*_*p*_ = 1, and the other is the background points, whose label value *l*_*p*_ = −1. For all the points, we initialize *θ*_*p*_ = 1. After completing the initialization, the algorithm goes into the iterative process and will not stop until there are not any pixels whose label will not change any more. Finally, pixels labelled 1 will belong to foreground and the one labelled with −1 will belong to background area. The iteration procedure is shown in Algorithm 1.

Here, *g* is a monotonous decreasing function bounded to [0, 1]. In the literature [15], the following energy function is taken:

(7) $$\begin{array}{c} g\left(x\right)=1-\frac{x}{\max{||\vec{C}||}_{2}}. \end{array}$$

The principle of GrowCut algorithm is simple and is High precise in image segmentation. However, there is a drawback in it, especially for medical image with fuzzy edges. That is its segmentation result usually accompanies with burr, which is hard to accept for medical image process, for doctors often diagnose diseases with the organ's smooth degree. In order to obtain a smooth segmentation result, the literature [15] proposes a patulous GrowCut approach, which adds two additional terms to the local transform function: one is that if a cell is surrounded with too many enemies and meets the condition that enemies^*t*^(*p*) ≥ *T*_1_, then it will be allowed to attack its neighbour cells; the other is that if the number of a cell's neighbour enemies meets the condition of enemies^*t*^(*p*) ≥ *T*_2_, it will be forcibly occupied by its weakest neighbour enemies, even if the strength of the enemy is weaker than the cell. The number of the enemies is defined as

(8) $$\begin{array}{c} \text{enemie}{\text{s}}^{t}\left(p\right)=\underset{l=1,K}{\max}\left(\sum_{q\in N\left(p\right),l_{q}^{t}\neq l_{p}^{t}}l\right). \end{array}$$

The thresholds *T*_1_ and *T*_2_ control the smooth of the edge. 

In GrowCut algorithm, the energy function is essentially important to the segmentation result. A common one shown in (7) is widely used in recent years. It only takes into account the gray difference between the seed point and its neighbourhood; so it can hardly make full use of the image information for high precise medical image segmentation. In this paper, a novel energy function is proposed to possess better performance than the traditional one. The new energy function is defined as

(9) $$\begin{array}{c} B_{\{p,q\}}=\exp\left(-\frac{{\left(I_{p}-I_{q}\right)}^{2}}{2\sigma^{2}}\right)\cdot \frac{1}{\mathrm{dist}\left(p,q\right)}. \end{array}$$

Here, *B* indicates the energy of any pixel and its neighbourhood in an image, *I*_*p*_ and *I*_*q*_ represent a pixel and its neighbour pixel, respectively, *σ* ^2^ is a covariance value between a pixel and its neighbour pixels, and dist⁡(*p*, *q*) is the distance of a pixel and its neighbour pixel.

The energy function (9) has two benefits: one is that it not only considers the gray difference between two pixels, but also takes into account the distance intense between the seed point and its neighbour pixels. The change degree of the seed point's neighbour pixels increases along with the increase of the *σ* ^2^. In the same way, we can get a strong competitiveness to accelerate the competing among germs and benefit to get a balance. The second value of the novel energy function is that it sets the competitiveness in inverse proportion to the distance between two pixels, which is more conforming to the real case of germ competition in the environment, and it is more beneficial to solve the problem of edge detection.

### 2.4. Image Segmentation Based on Snakes and the Improved GrowCut Algorithm

In this section, we will introduce our proposed liver image segmentation algorithm based on Snakes and the improved GrowCut approach and discuss why we choose Snakes model as the further segmentation method, but not just the improved GrowCut result. 

**Figure 4:**
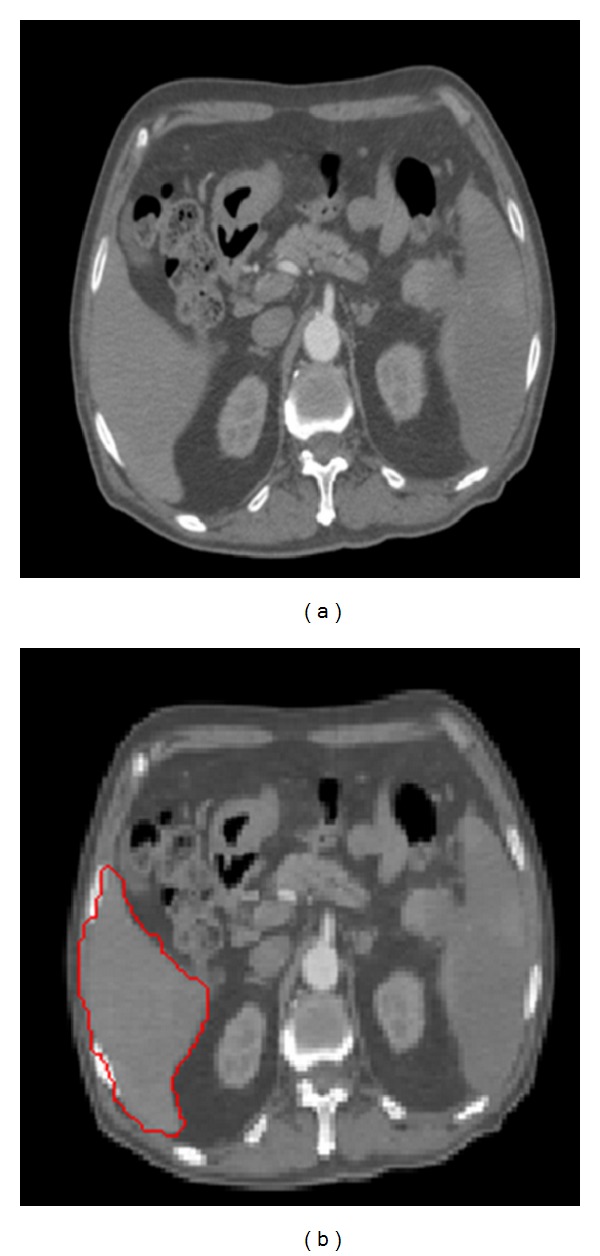
GrowCut segmentation results. (a) Original image; (b) result image of KIGC.

As shown in Figure 4, although a combination of K-means and the improved GrowCut (KIGC) can obtain the area of fuzzy edge, the edge is not smooth enough so we need to use Snakes model as the precise segmentation process method which takes the GrowCut result as initial contour.

Snakes model is a kind of active contours model [11], which owned a line with minimized energy. The line is constrained by both the external restrain force and the internal image force. It finally guides the curve to the feature region of an image, such as line or image edge, and accurately locks its neighbour boundary. 

The traditional Snakes model is a curve, which meets

(10) $$\begin{array}{c} x\left(s\right)=\left[x\left(s\right),y\left(s\right)\right],\;s\in \left[0,1\right]. \end{array}$$

This curve moves in the image space until the following equation is minimized:

(11) $$\begin{array}{c} E=\int_{0}^{1}\frac{1}{2}\left(\alpha {\left|x^{\prime }\left(s\right)\right|}^{2}+\beta {\left|x^{\prime \prime }\left(s\right)\right|}^{2}\right)+E_{\text{ext}}\left(x\left(s\right)\right)ds. \end{array}$$

Here, *α* and *β* are, respectively, the weight parameters of the opening degree and hardness, *x*′(*s*) and *x*′′(*s*) are, respectively, the first order derivative and the second-order derivative. The external energy of Snakes model comes from the image; so it should obtain a lesser value in the region of interest of an image and it should drag the curve to the edge of an image. A typical external energy function is shown as

(12) $$\begin{array}{c} E_{\text{ext}}\left(x,y\right)=-{\left|\nabla \left(G_{\sigma }\left(x,y\right)*I\left(x,y\right)\right)\right|}^{2} \end{array}$$

Here, *G*_*σ*_(*x*, *y*) is a 2D Gaussian function, whose root mean square deviation is *σ*. ∇ is a gradient operators. From (12), we can see that if the value of *σ* is too big, then the edge of an image is likely to be vague. We can get the iteration formula according to calculus of variations:

(13) $$\begin{array}{c} X_{\text{snake}}^{t+\Delta t}=M^{-1}\left(X_{\text{snake}}^{t}-\Delta t\frac{\partial E_{\text{ext}}}{\partial X_{\text{snake}}^{t}}\right)\\ Y_{\text{snake}}^{t+\Delta t}=M^{-1}\left(Y_{\text{snake}}^{t}-\Delta t\frac{\partial E_{\text{ext}}}{\partial Y_{\text{snake}}^{t}}\right). \end{array}$$

However, there is a serious drawback in the Snakes model - the attraction area of the external force is too limited, so that a perfect result can be obtained only if the initial curve is close to the edge of an image. To solve this drawback, there are many improved algorithms proposed by some experts. The most famous one is the Gradient Vector Flow (GVF) model proposed in literature [24–26], which begins with the calculation of a field of forces, called the GVF forces, and over in the image domain. The GVF forces are used to drive the Snakes, modelled as a physical object which has a resistance to both stretching and bending, towards the boundaries of the object. The GVF forces are calculated by applying generalized diffusion equations to both components of the gradient of an image edge map. The GVF model can be expressed as

(14) $$\begin{array}{c} w\left(x,y\right)=\left(u\left(x,y\right),v\left(x,y\right)\right). \end{array}$$

The minimized energy function is formulized as

(15) $$\begin{array}{c} E=\iint \left(u\left(u_{x}^{2}+u_{y}^{2}+v_{x}^{2}+v_{y}^{2}\right)+{\left|\nabla f\right|}^{2}{\left|w\left(x,y\right)-\nabla f\right|}^{2}\right)dx\;dy. \end{array}$$

Here, the function of the item *u*(*u*_*x*_^2^ + *u*_*y*_^2^ + *v*_*x*_^2^ + *v*_*y*_^2^) is to smooth an image, *u*_*x*_, *u*_*y*_, *v*_*x*_, *v*_*y*_ are, respectively, partial derivatives of *u*, *v* to *x* and *y*, and *u* is a regulative parameter. Based on the calculus of variations, we can get the following equation:

(16) $$\begin{array}{c} u\nabla^{2}u-\left(u-f_{x}\right)\left(f_{x}^{2}+f_{y}^{2}\right)=0\\ u\nabla^{2}v-\left(v-f_{x}\right)\left(f_{x}^{2}+f_{y}^{2}\right)=0. \end{array}$$

An image segmentation algorithm based on the Snakes model is proposed in this paper and applies with an improved GrowCut method to serve as a preprocess. The overall procedure of the proposed algorithm can be described as follows. Firstly, the improved GrowCut approach is used to segment an image roughly. Then, the edge of the above result is extracted and serves as an initial contour of the Snakes model. At Last, the liver region is obtained based on the Snakes model.

## 3. Experiments and Discussion

The experiments with liver CT images were carried out to demonstrate the performance of the proposed improved approach. The experimental data are 30 abdominal CT images with a format of DICOM derived from a 64 row CT machine in a domestic large hospital. The spatial resolution of each CT slice is 512 × 512. In order to improve the calculation speed, we convert the DICOM images into BMP images, whose gray level is 256. All experiments were conducted on a computer with Pentium processor of 3 GHz and memory of 1 GB.

### 3.1. Image Denoise Based on Wavelet Transform

In the denoise processing based on wavelet transform, there are mainly two factors that can affect the result of image denoise: one is wavelet base and the other is the denoise method. In this paper, several experiments were conducted based on different denoise methods. Figure 4 shows the results of different denoise methods using the same wavelet base which can be adjusted by users.

**Figure 5:**
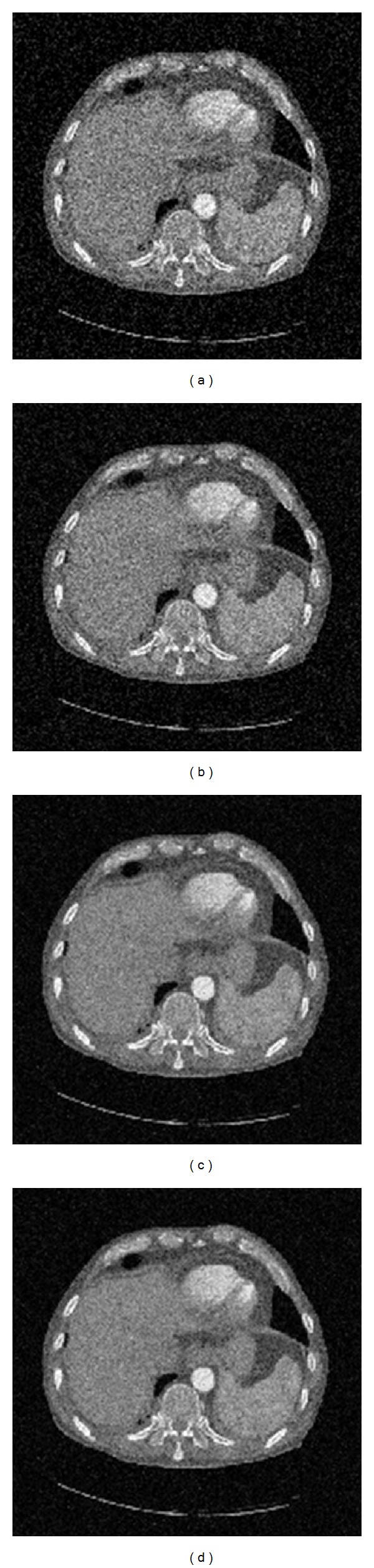
Results of image denoise based on different methods.

Figure 5(a) is the original image, Figure 5(b) is the denoised image by setting high frequency coefficients zero, Figure 5(c) is the denoised image using a fixed threshold for high frequency coefficients, and Figure 5(d) is the denoised image based on Bayesian threshold operation. The results show that Figure 5(d) is better than the other methods.

### 3.2. Image Segmentation Based on the Improved GrowCut Approach

**Figure 6:**
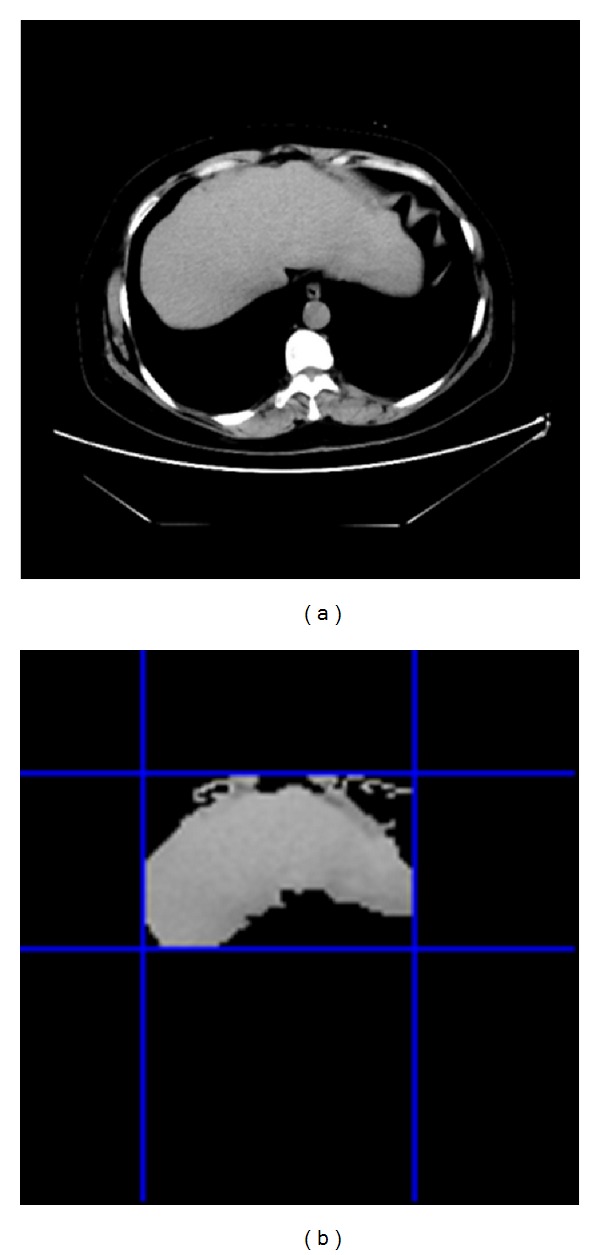
K-means clustering algorithm.

To prove the universality of K-means clustering algorithm, this paper gives a set of experimental results as shown in Figure 6. In Figure 6, we can see that K-means algorithm can effectively classify the liver's region of interest which not only improves the automaticity of selecting the target segmentation region but also benefits by improving the efficiency of the crude segmentation process.

**Figure 7:**
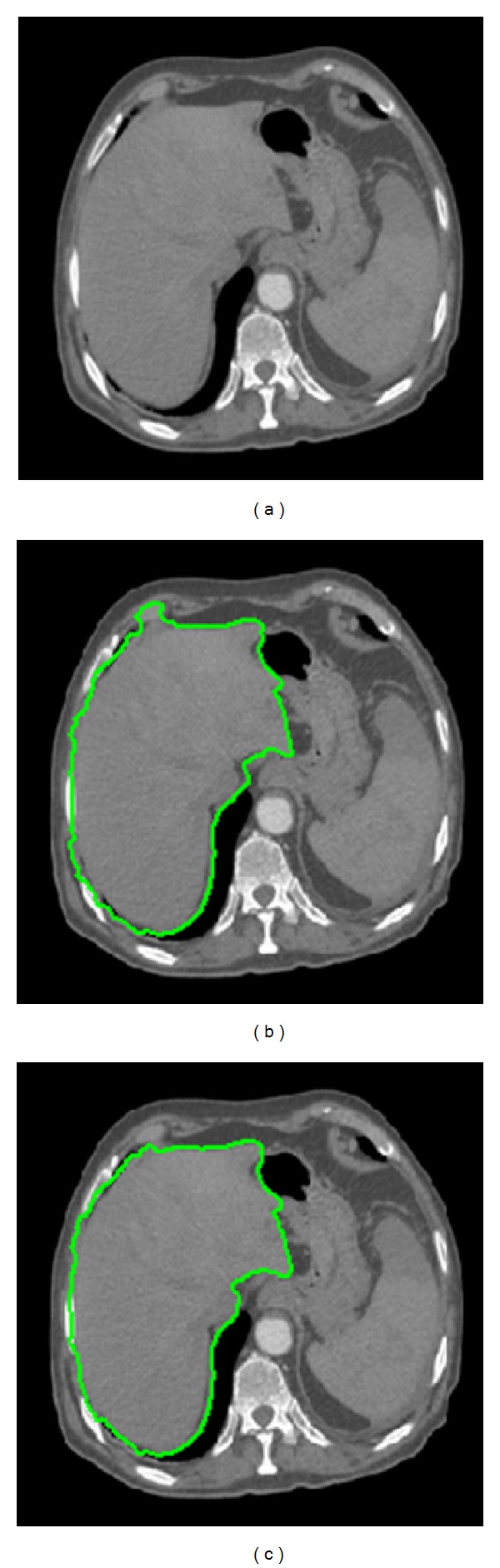
Comparisons between the tradition GrowCut and our improved approach.

Figure 7 gives the comparisons between the traditional GrowCut approach and our improved GrowCut approach. In Figure 7(a) is for the original images, Figure 7(b) is for the segmentation results based on the traditional GrowCut approach, and Figure 7(c) is for the segmentation results based on our improved algorithm.

As shown in Figure 7(b), the boundaries of regions are not very smooth, and many pixels around the left lobe are misclassified. However, the result of using the improved algorithm, as shown in Figure 7(c), demonstrates a visually significant improvement and robustness to noises. It also preserves better edge information than the traditional approach. The number of misclassified pixels is less than that of the traditional algorithm. However, there still exist under-segmentation problems in the left lobe of the liver, especially for some CT images with complicated organs.

**Figure 8:**
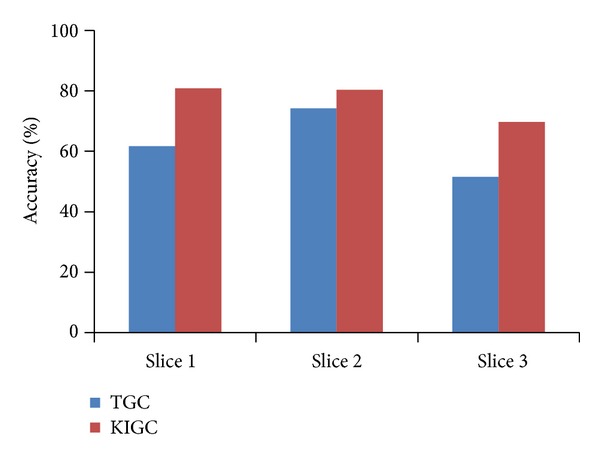
The accuracy comparison of TGC and KIGC.

To prove the effectiveness of the new energy function, we also compare the accuracy of the traditional GrowCut (TGC) method and a combination of K-means and the improved GrowCut (KIGC) quantitatively, as shown in Figure 8. The precision is computed using the following equation:

(17) $$\begin{array}{c} \text{precision}=\frac{2\times \left(S_{1}\cap S_{2}\right)}{S_{1}+S_{2}}, \end{array}$$

where *S*_1_ denotes the segmentation results and *S*_2_ is the manual segmentation results by a doctor.

In Figure 8, the accuracy is the average accuracy of each slice of 30 group image.

**Figure 9:**
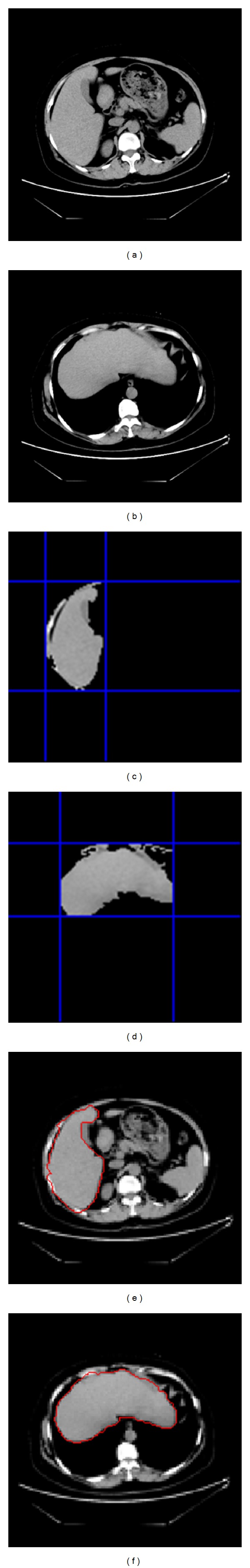
Comparisons of liver segmentation based on GrowCut and KIGC.

However, for some slices, our method does not improve the efficiency obviously. After K-means clustering, we can get a smaller liver boundary rectangle as an input to GrowCut segmentation. This can will reduce the bacterial ecological space of GrowCut and will greatly improve the efficiency. The traditional GrowCut and GrowCut based on K-means clustering image segmentation results are shown in Figure 9.

Figures 9(a) and 9(b) are two different liver images, Figures 9(c) and 9(d) are, respectively, the K-means clustering result image for the two images, and Figures 9(e) and 9(f) correspond to the segmentation result. When using the same marked points, traditional GrowCut and the one using K-means clustering obtain the same segmentation results. Although K-means-GrowCut method does not improve the accuracy, it can greatly reduce the segmentation time.

**Figure 10:**
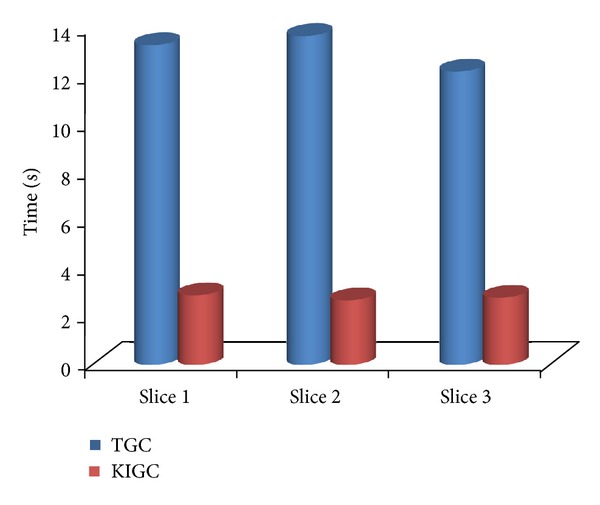
The comparison of segmentation time using and not using K-means.

To prove the efficiency, we compare the segmentation time of traditional GrowCut (TGC) and the improved GrowCut using K-means preprocessing (KIGC). As shown in Figure 10, the time is the average time of three slices of 30 group CT DICOM images. The final experiment results show that the segmentation time can be reduced greatly when we use K-means as a preprocessing method because of reducing other interference organs in abdomen CT image, as shown in Figure 6.

Table 1 gives a contrast result of segmentation time and spatial resolution between the traditional GrowCut and our improved GrowCut algorithm. It explains the effectiveness of using K-means clustering in the respect of specific spatial resolution. Although the improved GrowCut using the new energy function cannot improve the time efficiency, which is achieved mainly by using K-means clustering, we just compare the segmentation time of traditional GrowCut and the improved GrowCut not including the time of K-means clustering to prove how K-means has a great influence on the whole method's efficiency.

As shown in Table 1, the spatial resolutions of three initial images are all 128 × 128, and after a K-means cluster operation, the spatial resolution of the disjunctive rectangles are reduced to 70 × 58, 34 × 62, and 63 × 51 respectively. The time represents the interval from the start of human interactive operation selecting seed and background points to end of the stop of the iteration. The results also show that the improved GrowCut algorithm is much faster than the traditional GrowCut algorithm.

### 3.3. Image Segmentation Based on KIGC-Snake Algorithm

**Figure 11:**
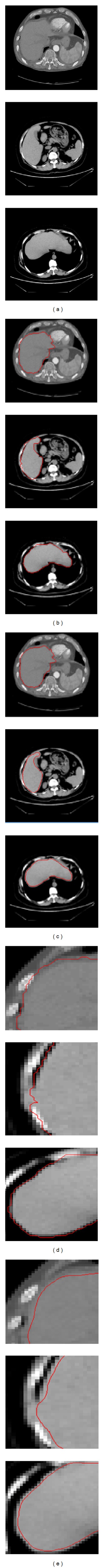
Segmentation results of two medical images based on the KIGC approach and KIGC-Snake algorithm.

Figure 11 gives three sets of the comparison experiment results.  Figure 11(a) is for the original images, Figure 11(d) is for the segmentation results based on the traditional GrowCut approach, Figure 11(c) is for the segmentation result of our proposed algorithm KIGC-Snake, Figure 11(d) is, respectively, the local magnified image of Figures 11(b), and 11(e) is, respectively, the local magnified image of Figure 11(c).

As shown in Figure 11, the segmentation results of our proposed algorithm in Figure 11(c), are much better than the results of KIGC approach in Figure 11(b). From Figures 11(d) and 11(e), we can see that the edges of our results are much better than the one not using Snakes model.

**Figure 12:**
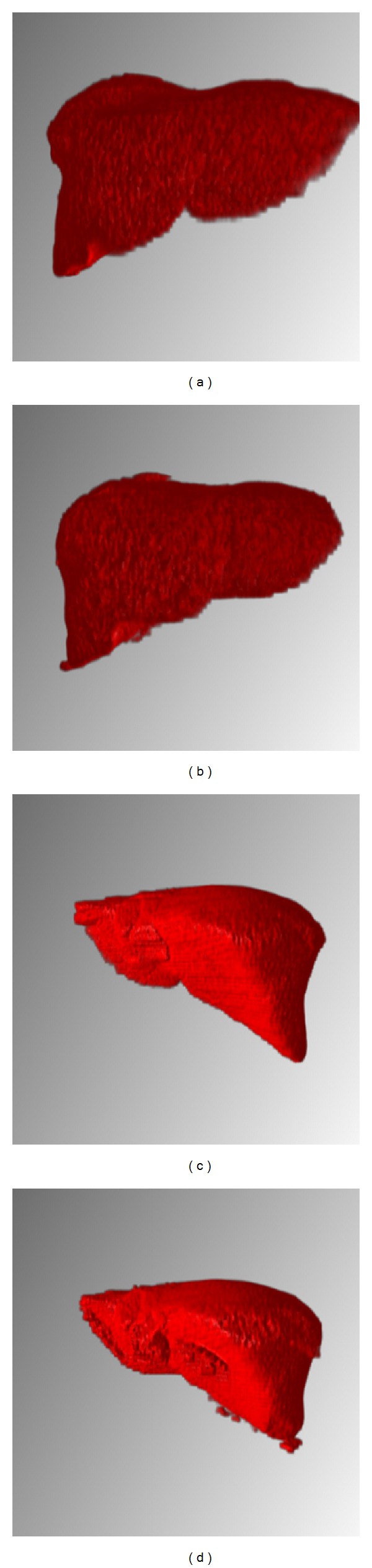
3D reconstruction results of our methods.

Figure 12 shows the liver three-dimensional reconstruction results based on our proposed method. Figures 12(a) and 12(c) are the three-dimensional reconstructions results of accurate liver segmentation. Figures 12(b) and 12(d) are the ones of the proposed method. Overall, compared with the accurate segmentation results (golden standard), the proposed method segmentation results are also good and there is no large error. From the detail view, comparing Figures 12(c) and 12(d) we can find that the proposed method can obtain a smooth liver three-dimensional model and other organizations' adhesions of its central location in the liver are very small. In contrast to Figures 12(a) and 12(b), our method has obvious segmentation problem in the position of the left hepatic lobe.

## 4. Conclusions

In this paper, a novel image segmentation algorithm based on the Snakes-GrowCut model is proposed for the liver segmentation in the abdominal CT images. According to the traditional GrowCut method, a new energy function is proposed based on graph theory to meet the high precise medical image segmentation, which not only considers the different intense between the seed point and its neighbour pixels, but also sets the competitiveness in inverse proportion to the distance between two pixels. Moreover, a pretreatment process through the K-means algorithm is conducted to reduce the running time. In additions, the multiple labels are taken in the improved GrowCut algorithm to get multiple organ segmentation results in a single operation. Lastly, Snakes model is used to conduct a precise segmentation by using the result obtained from the improved GrowCut approach. Several experiments with liver CT images are carried out to demonstrate the performance of the proposed improved approach. The experimental results show that the proposed approach not only has a better robustness and precision but also is much faster than the traditional GrowCut algorithm.

## Figures and Tables

**Algorithm 1:**
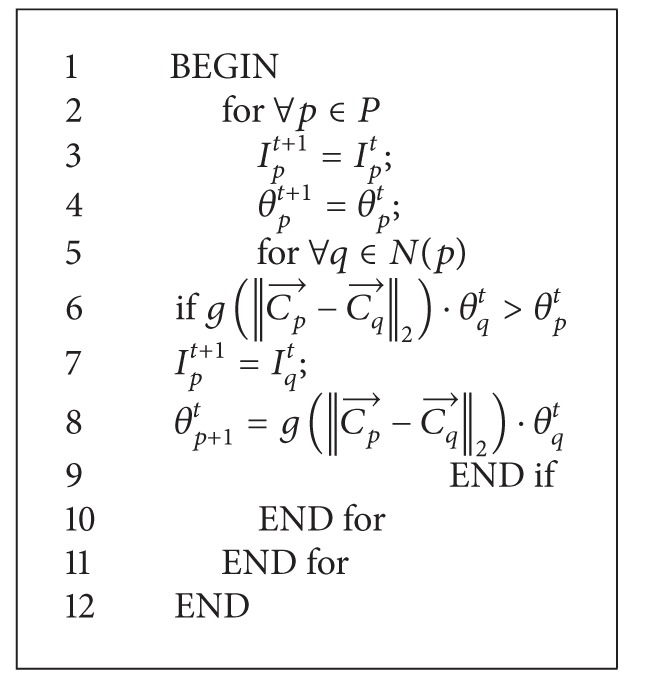
Function Automata evolution rule.

**Table 1:** Comparison of spatial resolution and segmentation time between the traditional GrowCut and our improved algorithm.

Data	Model	Spatial resolution	Time/s
Slice 1	GrowCut	128 × 128	12.94
	Improved GrowCut	70 × 58	0.78
Slice 2	GrowCut	128 × 128	13.23
	Improved GrowCut	34 × 62	0.46
Slice 3	GrowCut	128 × 128	11.88
	Improved GrowCut	63 × 51	0.71
